# Can Basic Medical Insurance Reduce Elderly Family Income Inequality in China?

**DOI:** 10.3389/fpubh.2022.838733

**Published:** 2022-02-15

**Authors:** Xiaohong Pu, Yilong Wang, Weike Zhang, Ming Zeng

**Affiliations:** ^1^School of Public Administration, Sichuan University, Chengdu, China; ^2^School of Management, Northwest Minzu University, Lanzhou, China

**Keywords:** basic medical insurance, elderly income inequality, Kakwani index, health performance, China

## Abstract

Basic medical insurance is the critical medical security system to realize common prosperity in China. This study explores the impact of basic medical insurance on elderly family income inequality in China using the China Family Panel Studies (CFPS) data in 2018. Our finding shows that basic medical insurance is significantly negatively correlated with elderly family income inequality, indicating basic medical insurance has a positive impact on narrowing the elderly family income inequality. The heterogeneity analysis shows that basic medical insurance has a more significant reduction effect among the eastern elderly and the younger elderly family. The results also suggest that health performance significantly mediates the relationship between basic medical insurance and elderly family income inequality. This study implies that the Chinese government should increase the proportion of basic medical insurance reimbursement and expand the scope of reimbursement for basic medical insurance to realize income fairness among elderly families.

## Introduction

China has been experiencing rapid aging over the past few decades. According to the seventh population census released by China's National Bureau of Statistics in 2021, the number of people aged 60 and above in China reaches 190 million by 2020, including 35.8 million aged 80 and above and accounting for 2.54 percent of the total population^1^. The World Health Organization predicts that by 2050, China will have 300 million people over 65 years old, accounting for 30 percent of the total population (1). An aging society will have a significant negative impact on China's economic growth and place a heavy burden on society as a whole in terms of the elderly family (2).

As the core of China's medical insurance system, basic medical insurance plays an essential role in preventing and resolving significant health risks and preventing sustained catastrophic medical and health expenditures (3). One of the fundamental stated goals of basic medical insurance is to reduce the impact of health shocks on family income and reduce poverty due to illness for the overall population, especially for the poor subgroups (4). Basic medical insurance is closely related to medical expenses and income distribution of the elderly family, and unreasonable and unfair medical insurance will aggravate elderly family income inequality (5). As the leading group vulnerable to diseases, the elderly spend a high proportion of their family income on medical care (6). Medical expenses and medical insurance are important factors affecting elderly family income^1^ inequality. In particular, concerning major diseases, the increased risk of disability and the high cost of post-care significantly impact elderly families, especially those with low income (7). Besides, the medical expenses of the elderly can affect the disposable income of the elderly and the whole family, and elderly family income inequality related to basic medical insurance can increase the risk of inter-generational income inequality transmission (8). Therefore, it is an exciting topic to explore whether basic medical insurance influences elderly family income inequality in China.

Several studies have found an association between basic medical insurance and income inequality. Specifically, some researchers have shown that basic medical insurance has a significant reduction effect on income inequality. For example, Fan et al. consider that the integration of social medical insurance policy has a positive impact on improving the rate of inpatient health services utilization. And both urban and rural catastrophic health expenditures all show a decreasing trend (9). Lu et al. report that UEBMI (Urban Employee Basic Medical Insurance) and URBMI (Urban Resident Basic Medical Insurance) significantly increase the monthly net income of agriculture migrants, especially in the lower-income group (10). In contrast, some researchers have demonstrated that basic medical insurance cannot significantly reduce income inequality. For example, Wang et al. suggest that URRBMI (Urban-Rural Resident Basic Medical Insurance) does not reduce the overall catastrophic health expenditures of residents effectively (11). Liu et al. suggest that there is no significant decrease in actual medical expenses after participating in basic medical insurance (12). Zhang et al. find that basic medical insurance is not essential in reducing income inequality among patients (13). So far, however, there has been little discussion about its impact on elderly family income inequality. Therefore, this study set out to explore the impact of basic medical insurance on elderly family income inequality in China.

This study uses the China Family Panel Studies (CFPS) data in 2018 to investigate how basic medical insurance affects elderly family income inequality in China. Besides, this study examines the mediation effect of health performance on the relationship between basic medical insurance and elderly family income inequality. The main contributions of this study are presented in three aspects. First, this study aims to contribute to this growing area of research by exploring the impact of basic medical insurance on elderly family income inequality. Second, this study analyzes the heterogeneous influence of basic medical insurance on elderly family income inequality from the perspectives of different regions and ages, and provides some important insights into elderly family income inequality. Finally, this study adopts the mediation effect model to examine the influence mechanism between basic medical insurance and elderly family income inequality through the mediating effect of health performance.

The remainder of this study is organized as follows. Section 2 proposes the research hypothesis. Section 3 presents the data sources and empirical models. Section 4 presents the empirical results and further analysis, and finally, Section 5 offers the research conclusions and policy recommendations.

## Research Hypothesis

For a long time, China's basic medical insurance system has suffered from systemic fragmentation and disparities between urban and rural areas (14), which results in the apparent “pro-rich” characteristics of the basic medical insurance system (3). In other words, the higher-income group can enjoy higher reimbursement rates and better quality medical treatment than the lower-income group (7). Since 2013, the Chinese government has carried out a series of medical insurance reforms to guarantee fairness for various income groups (15). For example, the Chinese government has integrated the basic medical insurance system for urban and rural residents to narrow the urban-rural differences in medical insurance (16). After the integration, basic medical insurance has effectively reduced the incidence of income poverty among the lower-income group and the widening income gap between urban and rural residents (17). Of course, basic medical insurance coverage can effectively reduce the catastrophic health expenditures among the elderly family, thus narrowing the reimbursement gap and the income gap between the high- and low-income of the elderly family (18). This effectively guarantees the fairness of basic medical insurance and can sufficiently reduce the elderly family income inequality.

Based on the above analysis, this study proposes the following hypothesis:

**H1:** Basic medical insurance can reduce elderly family income inequality in China.

Basic medical insurance policies vary from region to region, province to province, and even city to city (19). Compared with central and western regions, the eastern region of China is more economically developed, and the local governments provide more subsidies for basic medical insurance (20). In medical treatment for the elderly, the reimbursement rate is generally higher in the eastern regions than that in the central and western regions (21, 22). The economic development of the central and western regions as a whole is less developed than that of the eastern regions (23), and the financial subsidies for basic medical insurance are not as strong as that of the local governments in the eastern regions (24). In general, compared with the central and western regions, basic medical insurance has a more significant inhibitory effect on the family income inequality of the elderly in the eastern regions.

The life cycle theory explains the objective fact that the incidence of disease increases significantly with age. China has entered an aging society, and the aging population mainly concentrates on the younger elderly of 60–80 years old (25). While some in this group remain in good health, there are apparent gaps in health status (26). This group has become the largest user and beneficiary group of basic medical insurance (27). Compared to the general reliance on basic medical insurance to compensate for the cost of treatment for illnesses suffered by aged 80 and above, basic medical insurance has a more remarkable dampening effect on the widening income deprivation of the younger elderly family of 60–80 years old (28).

Based on the above analysis, this study proposes the following hypotheses:

**H2a:** The impact of basic medical insurance on elderly family income inequality in the eastern regions is more significant than in the central and western regions.
**H2b:** The impact of basic health insurance on the younger family income inequality is more significant than that of the advanced elderly family.

According to existing research, basic medical insurance has a positive effect on improving the health performance of the elderly (29). On the one hand, basic medical insurance can improve health awareness and health care knowledge of the elderly (30). On the other hand, basic medical insurance provides the elderly with protection against illness (31), which increases the enthusiasm of the elderly to seek medical care and prevents minor illnesses from becoming serious ones (32). Meanwhile, the improved health performance of the elderly is conducive to reducing elderly family income inequality. The underlying reason is that the elderly family with low income is more sensitive to health expenditures than the high-income elderly family. The improved health performance has led to a significant reduction in health expenditures and narrowing income gaps among elderly families (33). Hence, basic medical insurance may reduce elderly family income inequality by promoting health performance.

Based on the above analysis, this study proposes the following hypothesis.

**H3**: Health performance mediates the impact of basic medical insurance on elderly family income inequality in China.

## Data, Variables, and Empirical Model

### Data

This study set out to examine the impact of basic medical insurance on elderly family income inequality using the China Family Panel Studies (CFPS) in 2018. The CFPS data was launched in 2010, which tracks more than 16,000 respondents from different parts of China every 1 or 2 years. CFPS focuses on the economic and non-economic wellbeing of China's population and a number of research topics, including economic activity, educational outcomes, family relations and family dynamics, population migration, health, etc. It is a national, large-scale, multidisciplinary social tracking survey project.

In addition, we choose CFPS data from 2018 for empirical analysis because only CFPS data from 2018 contain detailed variables of basic health insurance, income, and health performance, which are the basis for this analysis. Following China's current statutory retirement age, 60 is used as the starting age for older adults. Hence, we select the samples aged over 60 and under 100 years. After removing the samples with missing values of key variables, we finally gain 4,521 samples.

### Variables

#### Dependent Variable

The dependent variable of this study is elderly family income inequality (*Inequality*). Most literature uses the relative deprivation index to reflect the degree of income inequality, which can be recognized as a suitable measuring method. This study uses the Kakwani index, an excellent relative deprivation index with dimensionless characteristics, to measure elderly family income inequality in China.

Referring to previous studies (34, 35), Kakwani index is defined as follows:


(1) $$\begin{array}{l} RD\left(x,x_{j}\right)=\;\frac{1}{nu_{X}}\overset{n}{\underset{i=j+1}{\sum }}\left(x_{i}-x_{j}\right)\;x_{1}<x_{2}<\ldots <x_{n} \end{array}$$

Where *RD*(*x, x*_*j*_) is the Kakwani index of a given elderly family *j*; *x* represents the elderly family income; *u*_*X*_ is the average elderly family income in all samples; and *n* represents the number of elderly families.

China's endowment pattern is mainly family-based, and the living conditions of the elderly are not entirely determined by their income but the family income (36). In other words, the life of the elderly after retirement is mainly supported by their children in China. Hence, we calculate the Kakwani index of the elderly using their total family income (*Inequality_tot*) and average family income (*Inequality_avg*). We take *Inequality_tot* as the main proxy variable of elderly family income inequality and adopt *Inequality_avg* to conduct our robust test.

#### Independent Variable

The independent variable of this study is basic medical insurance (*Insurance*). We assign 1 to *Insurance* if the elderly have basic medical insurance reimbursement, otherwise 0.

#### Control Variables

Referring to the previous literature (37–39), we control the following variables: gender (*Gender*), education level (*Educ*), marriage (*Marriage*), retirement (*Retirement*), political outlook (*Outlook*), household size (*Size*), cash deposits (*Cash*), life satisfaction (*Satisfaction*), happiness level (*Happiness*), medical condition (*Condition*), and internet use (*Internet*). All control variables and their definitions are shown in Table 1.

**Table 1:** The definitions of all variables.

**Variables**	**Symbols**	**Definitions**
**Dependent variable**		
Elderly family income inequality	Inequality_tot	The Kakwani index of the elderly based on total family income.
	Inequality_avg	The Kakwani index of the elderly based on average family income.
**Independent variable**		
Basic medical insurance	Insurance	*Insurance* is equal to 1 if the elderly have basic medical insurance reimbursement, and otherwise 0.
**Mediating variable**		
Health performance	Health	*Health* is equal to 1–3, representing unhealthy, fair, healthy, respectively.
**Control variables**		
Gender	Gender	*Gender* is equal 1 if the elderly are male, and otherwise 0.
Education level	Educ	*Educ* is 1–7, indicating that education is unschooling, primary school, middle school, high school, bachelor's, master's and doctor's degree, respectively.
Marriage	Marriage	*Marriage* is equal 1 if the elderly are in marriage period, and otherwise 0.
Retirement	Retirement	*Retirement* is equal 1 if the elderly are retiring, and otherwise 0.
Political outlook	Outlook	*Outlook* is equal 1 if the elderly are party member, and otherwise 0.
Household size	Size	*Size* represents the number of the elderly family members.
Cash deposits	Cash	*Cash* represents the logarithm of cash deposits for the elderly family.
Life satisfaction	Satisfaction	*Satisfaction* is equal to 1–5 scale, representing the elderly life satisfaction, respectively.
Happiness level	Happiness	*Happiness* is to 1–10 sacle, representing the elderly happiness level, respectively.
Medical condition	Condition	*Condition* is 1–5, representing the elderly medical conditions are very bad, bad, fair, good, and very good, respecitvely.
Internet use	Internet	Internet is equal 1 if the elderly are using internet, and otherwise 0.

#### Mediating Variable

This study aims to explore the mediating effect of health performance (*Health*) between basic medical insurance and elderly family income inequality. We measure the health performance of the elderly based on the question of “Health status.” Referring to the previous literature (40), we assign 1 to *Health* if the elderly answer unhealthy, 2 for fair, and 3 for healthy.

The above variables and their definitions are presented in Table 1, and their descriptive statistics are shown in Table 2.

**Table 2:** Descriptive statistics.

**Variables**	**Obs**	**Mean**	**S.D**.	**Min**	**Max**
Inequality_tot	4,521	0.54	0.26	0.01	1.00
Inequality_avg	4,521	0.56	0.23	0.00	1.00
Insurance	4,521	0.51	0.50	0.00	1.00
Gender	4,521	0.46	0.50	0.00	1.00
Educ	4,521	1.92	1.07	1.00	7.00
Marriage	4,521	0.82	0.38	0.00	1.00
Retirement	4,521	0.17	0.38	0.00	1.00
Outlook	4,521	0.01	0.07	0.00	1.00
Size	4,521	3.81	2.13	1.00	21.00
Cash	4,521	9.75	2.02	1.10	15.32
Satisfaction	4,521	4.23	0.92	1.00	5.00
Happiness	4521	7.74	2.20	0.00	10.00
Condition	4,521	2.49	0.67	1.00	3.00
Internet	4,521	0.03	0.16	0.00	1.00
Health	4,521	2.30	0.56	1.00	3.00

### Empirical Model

Referring to the previous literature (13, 38, 41), we construct the following model to test the impact of basic medical insurance on elderly family income inequality.


(2) $$\begin{array}{l} Inequality_{i}=\alpha +\beta Insurace_{i}+\delta Controls+\varepsilon_{i} \end{array}$$

where *Inequality* is the dependent variable of elderly family income inequality; *Insurance* is the independent variable of basic medical insurance; *Controls* represents the control variables, including *Gender, Educ, Marriage, Retirement, Outlook, Size, Cash, Satisfaction, Happiness, Condition*, and *Internet*; α denotes the intercepted item; β is the coefficient of interest; δ denotes the coefficients of the control variables; *i* represents the elderly individual, and ε_i_ is a normally distributed random error vector.

We run Formulas (2) based on the OLS regression. As we can see, β is the coefficient of the variable of interest. If β is smaller than 0, it indicates that the bigger *Insurance* is, the smaller *Inequality* is. In other words, basic medical insurance reduces elderly family income inequality. If so, H1 is verified by the data. In contrast, if β is bigger than 0, it indicates that basic medical insurance worsens elderly family income inequality. If so, H1 doesn't stand according to our data.

## Empirical Results and Analysis

### Benchmark Regression Results

In this section, we conduct the empirical analysis by using Model (1) and report the results in Table 3. Among them, Column (1) reports the results without control variables, and Column (2) reports the results with control variables. We can find that all the coefficients of *Insurance* are significant and negative at the 1% level. These results indicate that basic medical insurance can reduce elderly family income inequality in China and support our H1. In other words, basic medical insurance coverage can effectively reduce the catastrophic health expenditures among the elderly family, and narrow the reimbursement gap and the income gap between the high- and low-income of the elderly family, which effectively guarantees the welfare of the family.

**Table 3:** The benchmark regression result.

**Variables**	**(1)**	**(2)**
Insurance	−0.052^***^	−0.022^***^
	(0.008)	(0.006)
Gender		0.035^***^
		(0.006)
Educ		−0.027^***^
		(0.003)
Marriage		0.017**
		(0.007)
Retirement		−0.133^***^
		(0.008)
Outlook		0.004^***^
		(0.040)
Size		−0.043^***^
		(0.001)
Cash		−0.058^***^
		(0.001)
Satisfaction		0.003
		(0.003)
Happiness		−0.005^***^
		(0.001)
Condition		−0.006
		(0.004)
Internet		−0.104^***^
		(0.018)
Constant	0.564^***^	1.344^***^
	(0.006)	(0.023)
Observations	4,521	4,521
R-squared	0.010	0.487

*** *p ≤ 0.01; Standard errors are in parentheses*.

In terms of the control variables, most of the estimates are in agreement with theoretical expectations. Specifically speaking, the coefficients of *Gender, Marriage*, and *Outlook* are significantly positive at the traditional statistic levels, indicating that elderly family income inequality is more significant among males, married people, and party members. The coefficients of *Edu, Retirement, Size, Cash, Happiness* and *Internet* are all significantly negative at the traditional statistic levels. These results suggest that the elderly family income inequality can be narrowed as education levels rise. The number of cash deposits, household size, happiness level, and internet use can reduce elderly family income inequality.

### Robustness Test

Following the previous literature (7), we use *Inequality_avg* as the alternative measure of elderly family income inequality to conduct the robustness test. The results are presented in Columns (1,2) of Table 4. As can be seen, all the coefficients of *Insurance* are significantly negative at the 1% level, which indicates that basic medical insurance significantly lowers elderly family income inequality. This result is consistent with the above findings and further supports our conclusions.

**Table 4:** Results of robustness test and endogeneity test.

**Variables**	**Robustness test**		**Endogeneity test**	
	**(1)**	**(2)**	**(3)**	**(4)**
Insurance	−0.050^***^	−0.018^***^		−0.031 ^***^
	(0.007)	(0.005)		(0.048)
Institution			−0.022^***^	
			(0.006)	
Constant	0.590^***^	1.081^***^	1.344^***^	1.402^***^
	(0.005)	(0.020)	(0.022)	(0.030)
Control variables	No	Yes	Yes	Yes
Observations	4,521	4,521	4,521	4,521
R-squared	0.012	0.425	0.487	0.191
Phase I F-value			58.75	
DWH test *p*-value				0.000

*** *p ≤ 0.01; Standard errors are in parentheses*.

### Endogeneity Test

Although we control for many factors affecting elderly family income inequality, there is still an endogenous problem. Hence, we use the two-stage least square method (2 SLS) to minimize the impact of the endogenous problem. To carry out the 2 SLS method, we construct an instrumental variable (denoted as *Institution*), and set *Institution* as 1 to 5, representing general hospitals, specialized hospitals, specialized community health centers or rural health centers, community health service stations or village health offices, and clinics, respectively. In the first-stage regression model, *Insurance* is the dependent variable, and *Institution* is the independent variable. While in the second-stage regression model, the dependent variable is *Inequality_tot*, and the independent variable is the fitted value of *Insurance* in the first-stage regression.

Furthermore, we choose *Institution* as the instrumental variable of *Insurance* for the following reasons. First, the supervision level of medical institutions in China is different, and the elderly are likely to pay various medical expenses in various medical institutions. Thus, the payment of basic health insurance for the elderly is related to the institutions they visit. Second, the elderly's choice of medical institutions has no direct impact on the elderly family income inequality. Besides, the result of the F-value [see Column (3) of Table 4] shows that there is no weak instrumental variable problem with *Institution*. Hence, *Institution* is an appropriate instrumental variable for *Insurance*.

The results of the endogeneity test are presented in Column (4) of Table 4. The estimated coefficient of the fitted value of *Insurance* is significantly negative, which is consistent with the above findings and further suggests that the conclusion is still robust.

### Heterogeneity Analysis

This section examines the heterogeneous impact of basic medical insurance on elderly family income inequality from the differences of region and age. The regression results are reported in Table 5. Columns (1-3) are the results of eastern region, central region and western region, respectively. Column (4) is the results of younger elderly, and Column (5) is the results of advanced elderly.

**Table 5:** Results of heterogeneous analysis.

**Variables**	**Region difference**			**Age difference**	
	**Eastern**	**Central**	**Western**	**Younger elderly**	**Advanced elderly**

	**(1)**	**(2)**	**(3)**	**(4)**	**(5)**
Basic medical insurance	−0.027^***^	−0.015	−0.004	−0.023^***^	−0.013
	(0.008)	(0.010)	(0.012)	(0.006)	(0.028)
Control variables	Yes	Yes	Yes	Yes	Yes
Constant	1.369^***^	1.333^***^	1.240^***^	1.351^***^	1.233^***^
	(0.033)	(0.040)	(0.048)	(0.023)	(0.119)
Observations	2,159	1,258	1,104	4,317	204
R–squared	0.395	0.362	0.263	0.367	0.475

*** *p ≤ 0.01; Standard errors are in parentheses*.

For the region differences, as can be seen from Columns (1-3) in Table 5, the coefficient of *Insurance* for the eastern regions is significantly negative at the 1% level, while it is not significant for central and western regions. That is, basic medical insurance can reduce elderly family income inequality in the eastern regions, but it has no effect in the central and western regions. Hence, the findings confirm H2a. Economically developed areas are mostly located in the eastern regions, where the reimbursement of basic medical insurance for the elderly is higher, and the proportion of medical costs borne by the elderly is lower. However, the economic development of central and western regions lags behind that of the eastern regions, and there is a severe imbalance in regional development. The reimbursement of basic medical insurance for the elderly varies significantly among regions. Therefore, basic medical insurance can significantly reduce eastern elderly family income inequality, but this effect does not exist for the central and western regions.

In terms of the age difference, 60–80 years old is defined as the younger elderly, and 80–100 years old is defined as the advanced elderly. As can be seen from Columns (4) and (5) in Table 5, the coefficient of *Insurance* for younger elderly is significantly negative at the 1% level, while it is not significant for advanced elderly. These results indicate that basic medical insurance can reduce the family income inequality of the younger elderly, but it has no effect on the advanced elderly family and confirms H2b. These results may be explained by the fact that the current elderly population in China is mainly concentrated in the younger elderly group, which accounts for 70% of the total elderly people, and this group uses basic medical insurance more frequently and intensively than the advanced elderly. Another possible explanation for this might be that the significant health disparities between the younger and advanced elderly, basic medical insurance is more effective in reducing family income inequality among the younger elderly due to illness shocks.

### Mediating Effect of Health Performance

Health performance is directly related to the applications of basic health insurance and elderly family income inequality, which may affect the relationship between them. Therefore, we examine the mediating role of health performance on the relationship between basic health insurance and elderly family income inequality in China.

Most studies adopt the stepwise causal test proposed by Baron et al. to test the mediating effect. However, there may be insufficient sample number or non-normal sample distribution for the data obtained from social surveys due to the limitations of research conditions, and the mediation effect may be biased using the stepwise causal test (42). Therefore, this study uses the Bootstrap method proposed by Hayes (43) to test the mediating effect of health performance, and the model is constructed as follows:


(3) $$\begin{array}{lll} Health_{i} & = & \alpha +\beta_{1}Insurace_{i}+\delta Controls_{i}+\varepsilon_{i} \end{array}$$

(4) $$\begin{array}{l} Inequality_{i}=\alpha +\beta Insurace_{i}\;\text{+}\;\beta_{2}Health_{i}+\\ \;\delta Controls_{i}+\;\varepsilon_{i} \end{array}$$

where *Health* represents the mediator variable of health performance, and the other variables are the same with the Model (1). The Bootstrap method can directly test whether β_1_ × β_2_ is significantly different from 0. If its coefficient is significantly different from 0, indicating that health performance has a mediating effect between basic medical insurance and elderly family income inequality.

Table 6 reports the results of the mediation analysis. We can find that the indirect and direct effects of health performance are significant at the 1% level (the coefficients are −0.002 and −0.049, respectively), and the proportion of indirect effect is 4.1%. These results indicate that health performance partly mediates the relationships between basic medical insurance and elderly family income inequality in China and confirms H3. The possible reason is that the reimbursement of basic medical insurance promotes the timely access of the elderly to medical services and prevents the deterioration of their health status. Thus, it is beneficial to decrease the risk of medical expenses for major diseases of the elderly and ultimately reduce elderly family income inequality.

**Table 6:** Results of mediating effect.

**Types**	**Observed coefficient**	**Bootstrap standard error**	***P*-value**
Indirect effect	−0.002	0.001	0.012
Direct effect	−0.049	0.008	0.000
The proportion of indirect effect			4.1%

## Conclusion and Implications

This study has examined the influence of basic medical insurance on elderly family income inequality in China through using the CFPS data in 2018 and also discussed the heterogeneity of region and age differences and the mediating effect of health performance.

This study adds to the growing body of researches about the impact of basic medical insurance on elderly family income inequality. Our empirical results show that basic medical insurance significantly reduces elderly family income inequality in China. In other words, basic medical insurance reduces the burden of the elderly and their families through medical expenses reimbursement, thus reducing the family income inequality of the elderly. We also find that the impact of basic medical insurance on elderly family income inequality varies with the differences of region and age. Specifically, basic health insurance plays a more significant role in reducing elderly family income inequality in the eastern regions than in the central and western regions. Besides, compared with the advanced elderly, the effect of basic medical insurance on reducing younger elderly family income inequality is more pronounced. Finally, we provide additional evidence that health performance plays a mediating effect between the relationship between basic medical insurance and elderly family income inequality in China.

The above conclusions have several important policy implications for China to promote the realization of the goal of “fair medical insurance” and the common prosperity of the elderly during the Fourth 5-Year Plan period (44). As a crucial medical security system design in China, basic medical insurance has a series of tasks such as adjusting the income distribution gap, guaranteeing health rights, and maintaining health justice (45). This study proposes that basic medical insurance can reduce elderly family income inequality and play a key role in maintaining welfare utility, which aligns with the original intention of institutional design to ensure fairness and promote stability. However, considering the impact of basic medical insurance on medical expenses and the income distribution of the elderly family, it is necessary to re-examine the basic medical insurance system in China. First, the Chinese government should narrow regional disparities in basic medical insurance reimbursement and compensation to ensure the fairness and sustainability of basic medical insurance. Second, the Chinese government should further integrate the basic medical insurance for urban and rural residents and the basic medical insurance for urban employees to reduce elderly family income inequality caused by participating in different types of basic medical insurance. Finally, the Chinese government should improve the design of basic medical insurance policies. Specifically, the government should optimize the welfare policy of medical insurance, such as providing more medical subsidies to the advanced elderly, including physical examination into the category of medical reimbursement, reducing the threshold line or increasing the reimbursement ratio.

## Data Availability Statement

Publicly available datasets were analyzed in this study. This data can be found here: https://opendata.pku.edu.cn/dataverse/CFPS.

## Author Contributions

XP: writing—original draft, data collection, and literature search. YW: conceptualization, writing—original draft, and software. WZ: writing—reviewing and editing and supervision. MZ: data collection. All authors contributed to the article and approved the submitted version.

## Funding

This work was supported by the Key Project of National Social Science Foundation of China (17ASH009), the Planning Project of Gansu Province Philosophy and Social Science (2021YB027), the 2021 Innovation and Entrepreneurship Reform Funds in Northwest Minzu University (4), the Education Department Innovation Funds in Gansu Province (2020B-074), and the Fundamental Research Funds for central universities in Northwest Minzu University (31920190117).

## Conflict of Interest

The authors declare that the research was conducted in the absence of any commercial or financial relationships that could be construed as a potential conflict of interest.

## Publisher's Note

All claims expressed in this article are solely those of the authors and do not necessarily represent those of their affiliated organizations, or those of the publisher, the editors and the reviewers. Any product that may be evaluated in this article, or claim that may be made by its manufacturer, is not guaranteed or endorsed by the publisher.

## Footnotes

^1^Available online at: http://www.scio.gov.cn/xwbfbh/wqfbh/44687/45470/ (accessed May 11, 2021).

## References

[1] Sun J Lyu XY Lyu SJ Zhao R. The effect of social participation on income-related inequality in health outcome among Chinese older adults. Int Health. (2021) 13:80–8. 10.1093/inthealth/ihaa023 33443288 PMC7807233

[2] Jiang YY Zhao TH Zheng HT. Population aging and its effects on the gap of urban public health insurance in China. China Econ Rev. (2021) 68:101646. 10.1016/j.chieco.2021.101646

[3] Zhou Y Wushouer H Vuillermin D Guan XD Shi LW. Does the universal medical insurance system reduce catastrophic health expenditure among middle-aged and elderly households in China? A longitudinal analysis. Eur J Health Econ. (2021) 22:463–471. 10.1007/s10198-021-01267-3 33582893

[4] Teng HY Cao ZZ Liu JL Liu P Hua W Yang Y. Health status and burden of health care costs among urban elderly in China. Asia Pac J Public He. (2015) 27:61S−8S. 10.1177/1010539515571579 25673281

[5] Zhou Q Qin XZ Liu GG. Relative economic status and mental health among Chinese adults: evidence from the China health and retirement longitudinal study. Rev Dev Econ. (2020) 24:1312–32. 10.1111/rode.1269

[6] Sun J Yao NL Lyu SJ. The association between urban and rural resident basic medical insurance and depressive symptoms among Chinese middle-aged and older adults: Evidence from the China health and retirement longitudinal study. Int J Health Plan M. (2021) 36:1916–35. 10.1002/hpm.3305 34435389

[7] Ma C Song Z Zong QQ. Urban-rural inequality of opportunity in health care: evidence from China. Int J Environ Res Public Health. (2021) 18:7792. 10.3390/ijerph18157792 34360084 PMC8345655

[8] Sun J Lyu SJ. The effect of medical insurance on catastrophic health expenditure: evidence from China. Cost Effect Resour A. (2020) 18:10. 10.1186/s12962-020-00206-y 32127784 PMC7045636

[9] Fan XJ Su M Si YF Zhao YX Zhou ZL. The benefits of an integrated social medical insurance for health services utlization in rural China: evidence from the China health and retirement longitudinal study. Int J Equity Health. (2021) 20:126. 10.1186/s12939-021-01457-8 34030719 PMC8145815

[10] Lu XJ Wang Q Wei DS. Do health insurance schemes heterogeneously affect income and income distribution? Evidence from Chinese agricultural migrants survey. Int J Environ Res Public Health. (2020) 17:3079. 10.3390/ijerph17093079 32354163 PMC7246720

[11] Wang JH Zhu H Liu H Wu K Zhang X Zhao MM. Can the reform of integrating health insurance reduce inequity in catastrophic health expenditure? Evidence from China. Int J Equity Health. (2020) 19:49. 10.1186/s12939-020-1145-5 32245473 PMC7126184

[12] Liu H Zhu H Wang JH Qi XY Zhao MM Shan LH. Catastrophic health expenditure incidence and its equity in China:a study on the inital implementation of the medical insurance integration system. BMC Public Health. (2019) 19:1761. 10.1186/s12889-019-8121-2 31888591 PMC6937839

[13] Zhang ZY Wang JB Jin MJ Li M Zhou LT Jing FY. Can medical insurance coverage reduce disparities of income in elderly patients requiring long-term care? The case of the People's Republic of China. Clin Interv Aging. (2014) 9:771–7. 10.2147/CIA.S58771 24855346 PMC4020881

[14] Meng YY Han JQ Qin SQ. The impact of health insurance policy on the health of the senior floating-Evidence from China. Int J Env Res Pub He. (2018) 15:2159. 10.3390/ijerph15102159 30275379 PMC6210087

[15] Wang SH Lar GH. Public and commercial medical insurance enrollment rates of rural-to-urban migrants in China. Front public health. (2021) 9:749330. 10.3389/fpubh.2021.749330 34917573 PMC8669388

[16] Shi XJ Wang CY Zhong T. Can public medical insurance promote rural entrepreneurship? Evidence from China. Appl Econ. (2021) 53:4292–309. 10.1080/00036846.2021.1899118

[17] Zhu YS Li YY Wu M Fu HQ. How do Chinese people perceive their healthcare system? Trends and determinants of public satisfaction and perceived fairness, 2006-2019. BMC Health Serv Res. (2022) 22:22. 10.1186/s12913-021-07413-0 34983522 PMC8725557

[18] Li SL Yang YF. An empirical study on the influence of the basic medical insurance for urban and rural residents on family financial asset allocation. Front Public Health. (2021) 9:725608. 10.3389/fpubh.2021.725608 34458230 PMC8387677

[19] Xu Yj Zhang T Wang DL. Changes in inequality in utilization of preventive care services: evidence on China's 2009 and 2015 health system reform. Int J Equity Health. (2019) 18:172. 10.1186/s12939-019-1078-z 31711485 PMC6849223

[20] Qin M Zhuang Y Liu HY. Old age insurance participation among rural-urban migrants in China. Demorg Res. (2015) 33:1047–66. 10.4054/DemRes.2015.33.3716041089

[21] Han JQ Meng YY. Institutional differences and geographical disparity: the impact of medical insurance on the equity of health services utilization by the floating elderly population – evidence from China. Int J Equity Health. (2019) 18:91. 10.1186/s12939-019-0988-y 31200716 PMC6570924

[22] Zhou GL Jan S Chen M Wang ZH Si L. Equity in healthcare financing following the introduction of the unified residents' health insurance scheme in China. Health Policy Plann. (2021) czab124. 10.1093/heapol/czab124. [Epub ahead of print]. 34651170

[23] Qin YD Chen L Li JB Wu YY Huang SH. Greater inequalities in dental caries treatment than in caries experience:a concentration index decomposition approach. BMC Oral Health. (2021) 21:564. 10.1186/s12903-021-01935-z 34749711 PMC8573976

[24] Cui XD Chang CT. How income influences health: decomposition based on absolute income and relative income effects. Int J Environ Res Public Health. (2021) 18:10738. 10.3390/ijerph182010738 34682479 PMC8535401

[25] Chen MS Zhou GL Si L. Ten years of progress towards universal health coverage: has China achieved equitable healthcare financing? BMJ Glob Health. (2020) 5:e003570. 10.1136/bmjgh-2020-003570 33208315 PMC7677383

[26] Zhou WR Li W Ning YC Gong SM Song NA Zhu BM. Social isolation is associated with rapid kidney function decline and the development of chronic kidney diseases in middle-aged and elderly adults: findings from the china health and retirement longitudinal study (CHARLS). Front Med. (2021) 8:782624. 10.3389/fmed.2021.782624 34926526 PMC8674531

[27] Liu XF Yang F Cheng WW Wu YY Cheng J Sun WC. Mixed methods research on satisfaction with basic medical insurance for urban and rural residents in China. BMC Public Health. (2020) 20:1201. 10.1186/s12889-020-09277-1 32758210 PMC7409480

[28] Xin Y Ren XH. The impact of family income on body mass index and self-rated health of illiterate and non-illiterate rural elderly in China: evidence from a fixed effect approach. Front Public Health. (2021) 9:722629. 10.3389/fpubh.2021.722629 34604161 PMC8484635

[29] Kato H Goto R Tsuji T Kondo K. The effects of patients cost-sharing on health expenditure and health among older people: heterogeneity across income groups. Eur J Health Econ. (2021). 10.1007/s10198-021-01399-6. [Epub ahead of print]. 34779932 PMC9170661

[30] Wu H Liu YQ. Examining inequality in utilisation of health management services for the elderly in rural Henan China. BMC Health Serv Res. (2020) 20:758. 10.1186/s12913-020-05630-7 32807153 PMC7433128

[31] Su CW Huang SW Tao R Haris M. Does economic overheating provide positive feedback on population health? Evidence from BRICS and ASEAN countries. Front Public Health. (2021) 9:661279. 10.3389/fpubh.2021.661279 33816429 PMC8012809

[32] Wang YX Yang W Avendano M. Income-related inequalities in informal care: evidence from the longitudinal healthy longevity survey in China. J Gerontol B Psychol. (2021) 76:1691–6. 10.1093/geronb/gbab043 33705540 PMC8522470

[33] He YY Zhou LL Li JS Wu J. An empirical analysis of the impact of income inequality and social capital on physical and mental health–take China's micro-database analysis as an example. Int J Equity Health. (2021) 20:241. 10.1186/s12939-021-01560-w 34742299 PMC8571851

[34] Papanikolaou N. Tax progressivity of personal wages and income inequality. J Risk Financ Manag. (2021) 14:60. 10.3390/jrfm1402006034399312

[35] Mantovani D Pellegrino S Vernizzi A. A note on the maximum value of the Kakwani index. Empir Econ. (2020) 58:869–874. 10.1007/s00181-018-1524-6

[36] Wang LJ Beland D Zhang SF. Pension fairness in China. China Econ Rev. (2014) 28:25–36. 10.1016/j.chieco.2013.11.003

[37] Ma MY Tian WX Kang J Li YZ Xia Q Wang NS. Does the medical insurance system play a real role in reducing catastrophic economic burdan in elderly patients with cardiovascular disease in China? Implication for accurately targeting vulnerable characteristics. Globalization Health. (2021) 17:36. 10.1186/s12992-021-00683-7 33781274 PMC8006647

[38] Wang ZW Chen YC Pan TY Liu XD Hu HW. The comparison of healthcare utilization inequality between URRBMI and NCMS in rural China. Int J Equity Health. (2019) 18:90. 10.1186/s12939-019-0987-1 31200711 PMC6567429

[39] Zhou ZL Fang Y Zhou ZY Li D Wang D Li YL. Assessing income-related health inequality and horizontal inequality in China. Soc Indic Res. (2017) 132:241–56. 10.1007/s11205-015-1221-134682615

[40] Cui SC Yu YS Dong WZ Xu TK Huang YY Zhang XY. Are there gender differencs in the trajectories of self-rated health among chinese older adults? An analysis of the Chinese longitudinal healthy longevity survey (CLHLS). BMC Geriatr. (2021) 21:563. 10.1186/s12877-021-02484-4 34663221 PMC8522225

[41] Jiang H Zhao MM Tian GM Zhao ZH Ding D Yin M. Perceived effect of financial risk protection by the urban-rural resident basic medical insurance scheme: a mixed-methods study of rural residents in China. BMJ Open. (2021) 11:e047699. 10.1136/bmjopen-2020-047699 34667000 PMC8527163

[42] Baron RM Kenny DA. The moderator mediator variable distinction in social psychological – research - conceptual, strategic, and statistical considerations. J Pers Soc Psychol. (1986) 51:1173–82. 10.1037/0022-3514.51.6.1173 3806354

[43] Hayes AF Scharkow M. The relative trustworthiness of inferential tests of the indirect effect in statistical mediation analysis: does method really matter? Psychol Sci. (2013) 24:1918–27. 10.1177/095679761348017 23955356

[44] Bai P Tang YX Zhang WK Zeng M. Does economic policy uncertainty matter for healthcare expenditure in China? A spatical economic analysis. Front Public Health. (2021) 9:676778. 10.3389/fpubh.2021.673778 34017814 PMC8129179

[45] Sun TT Tao R Su C W Umar M. How do economic fluctuations affect the mortality of infectious diseases?. Front Public Health. (2021) 9:678213. 10.3389/fpubh.2021.678213 33968891 PMC8100195

